# A feasibility study exploring precarious employment and stress-related health among women

**DOI:** 10.1186/s12889-026-27366-5

**Published:** 2026-04-15

**Authors:** Vanessa M. Oddo, Sarah B. Andrea, Megan R. Winkler, Emily Q. Ahonen, Theo K. Bammler, Lisa M. Tussing-Humphreys, Anjum Hajat

**Affiliations:** 1 Department of Kinesiology and Nutrition, College of Applied Health Sciences, University of Illinois Chicago, 1919 W Taylor Street, MC 517, Chicago, IL 60612 USA; 2 University of Illinois Cancer Center, IL 60612, Chicago, USA; 3 School of Public Health, Oregon Health and Sciences University–Portland State University, Portland, Oregon USA; 4 Department of Behavioral, Social and Health Education Sciences, Rollins School of Public Health, Emory University, Atlanta, Georgia USA; 5 Division of Occupational and Environmental Health, School of Medicine, University of Utah, Salt Lake City, Utah USA; 6 Department of Environmental and Occupational Health Sciences, Interdisciplinary Center for Exposures, Diseases, Genomics and Environment, School of Public Health, University of Washington, Seattle, Washington USA; 7 Department of Epidemiology, School of Public Health, University of Washington, Seattle, Washington USA

**Keywords:** Employment quality, C-reactive protein, Cortisol, Biological stress, Social determinant of health, Chicago Illinois

## Abstract

**Background:**

Stress is a hypothesized mechanism through which precarious employment (PE) may contribute to poor health; however, there is a limited understanding of this potential mechanism in the U.S. This study aimed to (1) provide preliminary insights into the biological stress profiles of women experiencing varying levels of PE and (2) test the feasibility of at-home collection of stress biomarkers, c-reactive protein (CRP), and salivary cortisol, among women in Chicago, Illinois.

**Methods:**

We recruited 101 working-age women between August-October 2023. An online survey collected information on demographics, PE, and self-reported mental (Center for Epidemiologic Studies Depression Scale, Cohen’s Perceived Stress Scale) and physical (self-reported health, body mass index) health. We mailed home-collection kits to collect capillary blood (120 𝜇L) and saliva (3 samples on 2 workdays). We used 27 employment indicators to construct a multidimensional PE score (PES) (range:0–10) and estimated descriptive statistics overall and by tertile of PE.

**Results:**

We reached our recruitment target of ~ 50 women/month, and 70% returned the test kits. The sample was diverse in age (18–40 years: 57%, 41–64 years: 43%) and education ($$\:\le\:$$highschool: 11%, some college: 29%, $$\:\ge\:$$bachelor’s: 60%). Non-Hispanic (NH)-Black (33%) and Hispanic (36%) women were over-represented, compared to NH-White women (28%). More than half earned <$40,000, and the average PES was 3.6 (SE = 0.3). The most precariously employed women identified as NH-Black and Hispanic, had lower levels of educational attainment, experienced discrimination and had poorer self-reported physical and mental health. Those lost-to-follow-up between the online survey and biospecimen collection were lower-education, lower-income, more precariously employed, identified as Hispanic, and had poorer health. Biomarker patterns across PE tertiles were inconsistent; however, there was some suggestive evidence that the most precariously employed group may have lower recovery cortisol trends throughout the day, compared to the low and mid PE groups.

**Conclusions:**

We demonstrate the feasibility of at-home data collection of biospecimen samples among working women. Larger studies of PE and health are warranted.

**Supplementary Information:**

The online version contains supplementary material available at 10.1186/s12889-026-27366-5.

## Introduction

In the United States (U.S.), up to one-third [1, 2] of the workforce is estimated to be precariously employed, commonly characterized as the accumulation of unfavorable facets of employment quality such as insufficient wages, job instability, limited fringe benefits and rights and protections, coupled with imbalanced employer-worker power relations [3, 4]. Prior studies find associations between precarious employment (PE) and poorer physical and mental health [5–7]. However, little is known about the mechanisms through which PE affects health.

Non-Hispanic [NH] Black and Hispanic women are disproportionately experiencing PE in the U.S. [1, 6–8] and in turn, experience employment-related stressors, including inequalities in hiring practices [9], lower wages [10–12], lack of fringe benefits packages [13], and unstable and unpredictable work scheduling practices [14]. Thus, stress is one of the main hypothesized mechanisms through which PE may contribute to adverse health and health disparities. Psychosocial stressors can activate the hypothalamic-pituitary-adrenal axis resulting in the release of several hormones including cortisol, the autonomic nervous system, and inflammatory cytokines (e.g., C-reactive protein [CRP]) through a series of feedback loops and direct activation [15–17]. In turn, cortisol promotes the storage of abdominal fat [18], influences blood pressure regulation [19], and the development of insulin resistance [20, 21]. Prior studies also report elevated cortisol and inflammation markers in relation to chronic disease and depressive symptoms [22, 23] and associations between perceived stress and accelerated aging [24], which in turn, is associated with adverse health conditions [25–27].

While prior work has investigated associations between income and poverty and stress [28–30], PE also encompasses other adverse employment conditions, that interact with and reinforce each other, including instability, lack of benefits, power imbalance, and inconsistent scheduling; the accumulation of multiple adverse employment conditions could differentially affect stress physiology and contribute to poor health, beyond low wages alone. Yet, no existing large-scale study has been designed to look specifically at the association between the multidimensional PE construct and stress in the U.S. Moreover, existing studies of PE and health have relied on secondary data sources, which either have limited health outcomes or less comprehensive indicators of PE, hampering our understanding of any mechanisms through which PE affects health [8, 31].

At the same time, it remains challenging to recruit precariously employed workers and NH-Black and Hispanic individuals, broadly, as study participants. More traditional recruitment approaches in occupational health include recruitment from employee rosters from certain occupations (e.g., healthcare) or labor unions. However, the increasing precariousness of employment in the U.S. limit these approaches. Likewise, more common sampling approaches, like using survey research companies (e.g. Qualtrics panels), may not include many precariously employed individuals or adequate samples of NH-Black or Hispanic individuals. Additionally, lower participation rates among individuals who do not identify as NH-White threaten the generalizability of research findings broadly, and their past experiences of abuse by researchers, fear of harm, as well as time, costs, and logistics are barriers that often discourage study participation [32–35]. At-home sample collection, with minimally invasive sampling techniques, such as capillary blood sampling and salivary cortisol, offers a novel and practical approach to collecting biospecimen data among wider segments of the population, particularly among traditionally “harder to engage” populations [36, 37]. Moreover, mail-based collection of biospecimens may create opportunities to recruit a larger and more geographically diverse participant population [38, 39]. However, there is relatively limited evidence on the feasibility of such an approach [38–42].

Thus, the study aims were two-fold: (1) to provide preliminary insights into the biological stress profiles of women experiencing varying levels of PE and (2) to evaluate the feasibility of remote biospecimen collection. Together, these insights can guide future population-based studies that can help identify employment-related intervention points beyond income support (e.g., improving job predictability, access to paid leave), while also understanding the feasibility of remote biospecimen collection.

## Methods

### Study design and participants

The primary focus of this study was to assess the feasibility of recruiting and collecting survey and biological data among ~ 100 employed women in the Chicago metropolitan area of Cook County, Illinois, USA; given that this was a feasibility study, Cook County (versus only Chicago) was selected to ensure that recruitment targets were met. The online survey queried women on demographic information, indicators of PE, and self-reported health indicators. Home-collection kits, featuring a novel blood collection method, were mailed to participants to collect biological samples. Measures are described in more detail below.

We employed a multi-pronged approach to recruit participants with a range of employment quality between August 10, 2023, and October 16, 2023. Recruitment approaches included targeted advertisements placed on Facebook (e.g., women with an associates degree or lower, in certain occupations [e.g. cleaning, care giving, service], and those working for specific companies [e.g., Instacart, McDonalds, Walmart, Amazon, Uber, Lyft, Jewel Osco]), health registries (University of Illinois Health, The New Normal), email listservs (e.g., Center for Healthy Work), community partnerships (Working Family Solidarity, Metropolitan Family Services), the distribution of flyers (e.g., grocery stores, laundromats), and referrals from current participants. Among those participants who indicated how they were recruited (*N* = 61), the majority were recruited via Facebook (~ 46%), followed by email listservs (~ 30%), flyers (~ 10%), community partnerships (~ 8%) and other methods (~ 6%).

Potential study participants were directed to an online form in Qualtrics where they completed screener questions to determine eligibility. Inclusion criteria included: (1) being working age (18 to 64 years); (2) speaking and reading English; (3) residing in Cook County, Illinois; (4) self-identifying as a woman; and (5) being currently employed or recently employed, defined as working for pay at least 3 months (at 10 hours/week) within the last 6 months (could be intermittent). Women were excluded if they were pregnant or had taken corticosteroids in the month prior due to their effect on the biomarkers collected. Additionally, we employed quotas by race/ethnicity ($\:\le\:$ 50% NH-White) and education ($\:\le\:$ 60% bachelor’s degree) to ensure a sample that included women with a range of employment quality. Qualifying individuals provided electronic consent to participate. All procedures were approved by the institutional review board at the University of Illinois Chicago.

### Study procedures

Following written consent, qualifying women were immediately directed to complete an online survey, which was administered and stored electronically via Qualtrics. Fraudulent responses were flagged, and removed, using a variety of Qualtrics built-in features such as duplicate responses and bot detection (i.e., reCAPTCHA scores). The survey took 17 minutes on average to complete. Following the completion of the online survey, participants received $20 via ClinCard e-giftcard .

Home-collection kits were then mailed to participants to collect capillary blood and saliva. Those with a recent vaccination or infection (e.g., cold/flu) were asked to refrain from saliva or blood collection until 1-week after they received their vaccine(s) or the infection has passed. To collect 120 𝜇L of capillary blood, we used the Mitra™ microsampling device based on VAMS^®^, which enables a volumetric absorptive approach to microsampling (Neoteryx, Trajan Scientific and Medical, four 30 𝜇L tips to get 120 𝜇L) and has previously been used in research settings [40]. The Mitra device is similar to dried blood spot technology, with several advantages; they can be easily shipped, do not require cold chain storage, and collect reasonably precise volumes at home. Prior studies also suggest the Mitra™ device is preferred by participants over dried blood spots or venous blood draws [43] and are higher quality than those taken in the clinical setting, which is attributed to lower anxiety [41]. In addition to the Mitra™ device, participants received lancets, gauze, bandages, a specimen bag with desiccant, a return shipping envelope, and step-by-step written instructions. Participants were instructed to prick their finger with a lancing device and place the tip of the Mitra™ device next to the blood spot. Typically, individuals can obtain sufficient blood samples to fill four 30 𝜇L tips with one finger prick/one lancet. However, additional lancets were provided in case participants were not able to obtain a sufficient sample of blood to fill the tips on the first try.

Home collection kits also included six collection tubes and cotton swabs (Salivette™) for the collection of salivary cortisol (three samples on two consecutive workdays). Participants were instructed to collect saliva samples by sliding the cotton swab from the plastic tube into the mouth and moving it from side to side for ~ two minutes at three time points (1) immediately after waking but before getting out of bed, (2) 30-minutes after waking, and (3) before bed. Participants were provided written instructions with images and asked to record the collection time of the salivary samples.

After the blood and saliva were collected, participants were instructed to ship both samples to the University of Washington’s Interdisciplinary Center for Exposures, Diseases, Genomics and Environment Lab with a desiccant and sealed within a pre-labeled postage-provided envelope. Participants were instructed to return samples within 2-weeks of receiving the collection kits. Our follow-up protocol was designed to help minimize attrition from the online survey to returning the home-collection kits. We first sent a “welcome” email to participants, three reminder emails, and one reminder text to participants over a one-month period. Upon completion of the home-collection kit, participants received an additional $50 for their participation via ClinCard. Additionally, for participants who did not return the biospecimen samples within three weeks, we offered an additional $10 incentive (via reminder email ). Thus, participants could receive a total of $80 for completing both the survey and biospecimen collection. The average time between completing the online survey and the lab receiving the samples was 26.4 days (standard deviation [SD]: 14.1).

### Survey measures

To assess the feasibility of reaching our desired population, we additionally developed and administered a survey that queried respondents on demographic and household information, indicators of PE, self-reported physical and mental health, food and nutrition security, and relevant medication usage (e.g., diabetes, anti-inflammatory drugs). Questions on demographic and household characteristics were derived from the NIH PhenX toolkit on the social determinants of health, and PE questions were largely derived from the Employment Precarity Index [44] and prior nationally-representative surveys in the U.S. Food security was measured using the two-item Hunger Vital Sign™ screener; responses in the affirmative [i.e., often sometimes] to either question were coded as food insecure (= 1) versus food secure (= 0). Nutrition security was measured using the validated one-item measure developed by the Center for Nutrition & Health Impact [45]. Responses in the affirmative (i.e., often or always) to the question: “In the last 30 days, we worried that the food we were able to eat would hurt our health and well-being” were assigned a 1 (versus 0).

Aligned with our hypothesis that PE contributes to adverse health through stress pathways, participants’ self-perceived level of stress, measured on a continuous scale, was assessed using the 10-item Cohen’s Perceived Stress Scale (PSS, possible range 0–50 with 0–13 = low, 14–26 = moderate, 37–40 = high) [46, 47]. Additionally, given that prior studies have report elevated cortisol and inflammation markers in relation to depressive symptoms [22, 23] we administered a standard screener questionnaire for depressive symptoms (Center for Epidemiologic Studies Depression Scale [CESD], range 0–40); depression severity was dichotomized as no or mild (0–9) versus moderate or severe [10–40, 48]. Physical health was assessed using self-reported height and weight, from which we calculated body mass index ([BMI]: kg/m^2^) and the question “in general, would you say your health is” with the response items including excellent, very good, good, fair, or poor; self-reported health (SRH) was dichotomized as excellent, very good, good (= 0) versus fair or poor (= 1).

### Biological measures

Our primary biological measures collected via the home kits included CRP (continuous) and cortisol (wake-up values, cortisol awakening response [CAR] and wake-to-bed slope) as these are commonly used markers of stress that are sensitive to psychosocial exposures [49, 50]. Because the lab received the blood specimens in four 30 𝜇L collection tips, no further aliquoting was necessary. Specimens were assayed once all specimens were received to avoid batch effects. We followed the manufacturer’s recommended protocol to extract the blood samples. The eluted blood samples were stored frozen at − 80 °C until analyzed, whereby the extracts were thawed and spun down for 10 seconds. All measurements were adjusted for total protein concentration of each sample; the Pierce BCA Protein Assay Kit (Thermo Fisher Scientific, Waltham, MA) was used to determine total protein concentrations according to the manufacturer’s recommended protocol. CRP was measured in duplicate with the Meso Scale Discovery (MSD) V-Plex assay with an electrochemiluminescence detection system (MSD Multiplex Platform, Sector Imager 2400, Meso Scale Discovery, Gaithersburg, Maryland). Standard curves were generated using purified analytes and MSD Discovery Workbench Software (Meso Scale Discovery, Gaithersburg, Maryland) to determine analyte concentrations of the samples. Cortisol was measured in duplicate with the Cortisol Saliva ELISA assay kit from Salimetrics^®^ (Salimetrics, Carlsbad, CA) according to the manufacturer’s recommended protocol.

### Precarious employment score

Using 27 employment indicators (see Appendix Table 1) we constructed a precarious employment score (PES) for each respondent. Similar to our prior work (e.g. [1, 5, 6]), the PES is a multidimensional construct, which can be characterized as the accumulation and interaction of unfavorable facets of employment quality based the following dimensions: material rewards (e.g., income), employment stability (e.g., job tenure), workers’ rights and social protections (e.g., paid overtime), working time arrangements (e.g., hours worked), collective organization (e.g., union coverage), and interpersonal power relations (e.g., ability to take leave) [51, 52]. An aggregate PES was created using a data-driven principal components analysis. Detailed in Appendix Table 2, material rewards (income, paid for time off, income variation, fringe benefits); schedule control, and interpersonal relations were the most influential variables in this sample-specific score. A higher PES (range: 0–10) reflected more precarious employment. We then created data-driven tertiles of PE (most precarious, mid precarious, least precarious).

**Table 1:** Selected sample characteristics

	Overall [*N* = 94]		Completed Blood Collection Kit [*N* = 60]		Not Completed Collection Kit [*N* = 34]	
	*N* or Mean	% or SE	*N* or Mean	% or SE	*N* or Mean	% or SE
Demographics						
Age						
18–30	25	26.6%	15	25.0%	10	29.4%
31–40	28	29.8%	18	30.0%	10	29.4%
41–50	22	23.4%	13	21.7%	9	26.5%
51–64	19	20.2%	14	23.3%	5	14.7%
Marital Status^1^						
Married	27	29.7%	18	30.0%	9	29.0%
Divorced/Separated	17	18.7%	13	21.7%	4	12.9%
Never Married	47	51.6%	29	48.3%	18	58.1%
Education						
≤ High School	10	10.6%	5	8.3%	5	14.7%
Some College or associates	27	28.7%	13	21.7%	14	41.2%
Bachelors	38	40.4%	27	45.0%	11	32.4%
Graduate	19	20.2%	15	25.0%	4	11.8%
Race/Ethnicity						
Non-Hispanic White	26	27.7%	19	31.7%	7	20.6%
Non-Hispanic Black	31	33.0%	20	33.3%	11	32.4%
Non-Hispanic Other	3	3.2%	3	5.0%	0	0.0%
Hispanic	34	36.2%	18	30.0%	16	47.1%
Income						
<$20,000	26	27.7%	16	26.7%	10	29.4%
$20,000–39,999	25	26.6%	14	23.3%	11	32.4%
$40,000–59,999	24	25.5%	12	20.0%	12	35.3%
$60,000–79,999	11	11.7%	10	16.7%	1	2.9%
$80,000+	8	8.5%	8	13.3%	0	0.0%
Located in Chicago	65	69.2%	41	68.3%	24	70.1%
US Born^1^	77	83.7%	49	81.7%	28	87.5%
Mean PES^2^	3.6	0.3	3.2	0.4	4.2	0.5
Nutrition Outcomes						
Food Insecure^3^	34	36.2%	19	31.7%	15	44.1%
Nutrition Insecure^4^	37	36.2%	22	36.7%	15	44.1%
Health Outcomes						
Mean PSS^5^	20.4	0.6	20.3	0.8	20.5	1.0
Severe Depressive Symptoms^6^	53	56.4%	33	55.0%	20	58.8%
Fair/Poor Self-Reported Health^7^	36	38.3%	17	28.3%	19	55.9%
Body Mass Index	31.4	0.8	30.1	1.0	34.0	1.3
Mean C-reactive protein [mg/L]	NA	NA	1.3	0.2	NA	NA
Mean Salivary Cortisol [nmol/L]^8^	NA	NA	9.0	0.5	NA	NA

*NA * Not applicable, *PES * Precarious employment score, *PSS * Perceived stress score, *SE* Standard Error

^1^ missing values: *n* = 3 marital status, *n* = 2 nativity

^2^ The PES was created using 27 employment indicators and principal components analysis

^3^ Food insecurity was defined using the two-item Hunger Vital Sign™ screener . Responses in the affirmative [i.e., often or sometimes, versus never] were assigned a 1 [versus 0]

^4^ Nutrition insecurity was queried using the one-item measure developed by the Center for Nutrition & Health Impact. Nutrition insecurity was defined as responding sometimes, often, or always to the question, “In the last 30 days, I worried that the food I was able to eat would hurt my health and well-being”

^5^ Assessed using the 10-item Cohen’s Perceived Stress Scale [PSS, possible range 0–50]

^6^ Assessed using the Center for Epidemiologic Studies Depression Scale [possible range 0–40]; depression severity was dichotomized as no or mild [0–9] versus moderate or severe [10–40]

^7^ Assessed using the one-item question “in general, would you say your health is” with the response items including excellent, very good, good, fair, or poor; self-reported health was dichotomized as excellent, very good, good [= 0] versus fair or poor [= 1]

^8^ Salivary cortisol *n* = 43

**Table 2:** Sample and self-reported health characteristics by tertile of precarious employment^1^

	Most Precarious [*N* = 31]		Mid Precarious [*N* = 31]		Least Precarious [*N* = 32]	
	*N* or Mean	% or SE	*N* or Mean	% or SE	*N* or Mean	% or SE
Demographics						
Age						
18–30	9	29.0%	7	22.6%	9	28.1%
31–40	7	22.6%	11	35.5%	10	31.3%
41–50	10	32.3%	6	19.4%	6	18.8%
51–64	5	16.1%	7	22.6%	7	21.9%
Marital Status^2^						
Married	9	31.0%	8	26.7%	10	31.3%
Divorced/Separated	5	17.2%	5	16.7%	7	21.9%
Never Married	15	51.7%	17	56.7%	15	46.9%
Education						
≤ High School	10	32.3%	0	0.00%	0	0.00%
Some College or associates	9	29.0%	12	38.7%	6	18.8%
Bachelors	8	25.8%	13	41.9%	17	53.1%
Graduate	4	12.9%	6	19.4%	9	28.1%
Race/Ethnicity						
Non-Hispanic White	3	9.7%	8	25.8%	15	46.9%
Non-Hispanic Black	16	51.6%	12	38.7%	3	9.4%
Non-Hispanic Other	1	3.2%	0	0.0%	2	6.3%
Hispanic	11	35.5%	11	35.5%	12	37.5%
Income						
<$20,000	22	71.0%	4	12.9%	0	0.0%
$20,000–39,999	5	16.1%	12	38.7%	8	25.0%
$40,000–59,999	4	12.9%	9	29.0%	11	34.4%
$60,000–79,999	0	0.0%	3	9.7%	8	25.0%
$80,000+	0	0.0%	3	9.7%	5	15.6%
US born^2^	23	76.7%	29	83.7%	25	83.7%
Mean PES^1^	7.1	0.3	3.0	0.2	0.7	0.1
Discrimination						
Experience due to race/ethnicity	7	22.6%	3	9.7%	3	9.4%
Experience due to gender	5	16.1%	4	12.9%	9	28.1%
Experience due to age	3	9.7%	4	12.9%	3	9.4%
Experience due to immigration	1	3.2%	0	0.0%	0	0.0%
Barrier for advancement opportunities	8	25.8%	7	22.6%	7	21.9%
Nutrition and Self-Reported Health						
Food Insecure^3^	18	58.1%	9	29.0%	7	21.9%
Nutrition Insecure^4^	14	45.2%	13	41.9%	10	31.3%
Mean PSS^5^	23.7	1.0	18.9	1.1	18.6	0.9
Severe Depressive Symptoms^6^	25	80.6%	15	48.4%	13	40.6%
Fair/Poor Self-Reported Health^7^	16	51.6%	10	32.3%	10	31.3%
BMI	33.9	1.6	32.3	1.2	28.3	1.3

*BMI * Body mass index, *PES * Precarious employment score, *PSS * Perceived stress score, *SE* Standard Error

^1^ The PES was created using 27 employment indicators and principal components analysis, and then PE tertiles were created

^2^ Missing values: *n* = 3 marital status, *n* = 2 nativity

^3^ Food insecurity was defined using the two-item Hunger Vital Sign™ screener e. Responses in the affirmative [i.e., often or sometimes, versus never] were assigned a 1 [versus 0]

^4^ Nutrition insecurity was queried using the one-item measure developed by the Center for Nutrition & Health Impact. Nutrition insecurity was defined as responding sometimes, often, or always to the question, “In the last 30 days, I worried that the food I was able to eat would hurt my health and well-being”

^5^ The PSS is based on the 10-item Cohen’s Stress Scale [range 0–50]

^6^ Assessed using the Center for Epidemiologic Studies Depression Scale [possible range 0–40]; depression severity was dichotomized as no or mild [0–9] versus moderate or severe [10–40]

^7^ Assessed using the one-item question “in general, would you say your health is” with the response items including excellent, very good, good, fair, or poor; self-reported health was dichotomized as excellent, very good, good [= 0] versus fair or poor [= 1]

### Statistical analyses

This feasibility study was not powered to test for associations; therefore, the data presented are descriptive [53]. Data were examined for outliers, and we estimated means and standard errors (SE) for continuous variables and frequencies for categorical variables overall and by tertile of PE. We also examined whether the return of the home-collection kits varied across demographics and other completed survey measures. Finally, as an exploratory analysis, we used unadjusted linear mixed-effects models, with a random intercept and unstructured correlation structure, to estimate the average wake-up values for cortisol, CAR, and wake-to-bed slope of cortisol by tertile of PE.

Analyses were performed using Stata software (version 16.1, College Station Texas). The procedures followed in this pilot study were in accordance with ethical standards for human research and approval was obtained from the Institutional Review Board of the University of Illinois Chicago.

## Results

### Recruitment and eligibility screening

**Fig. 1:**
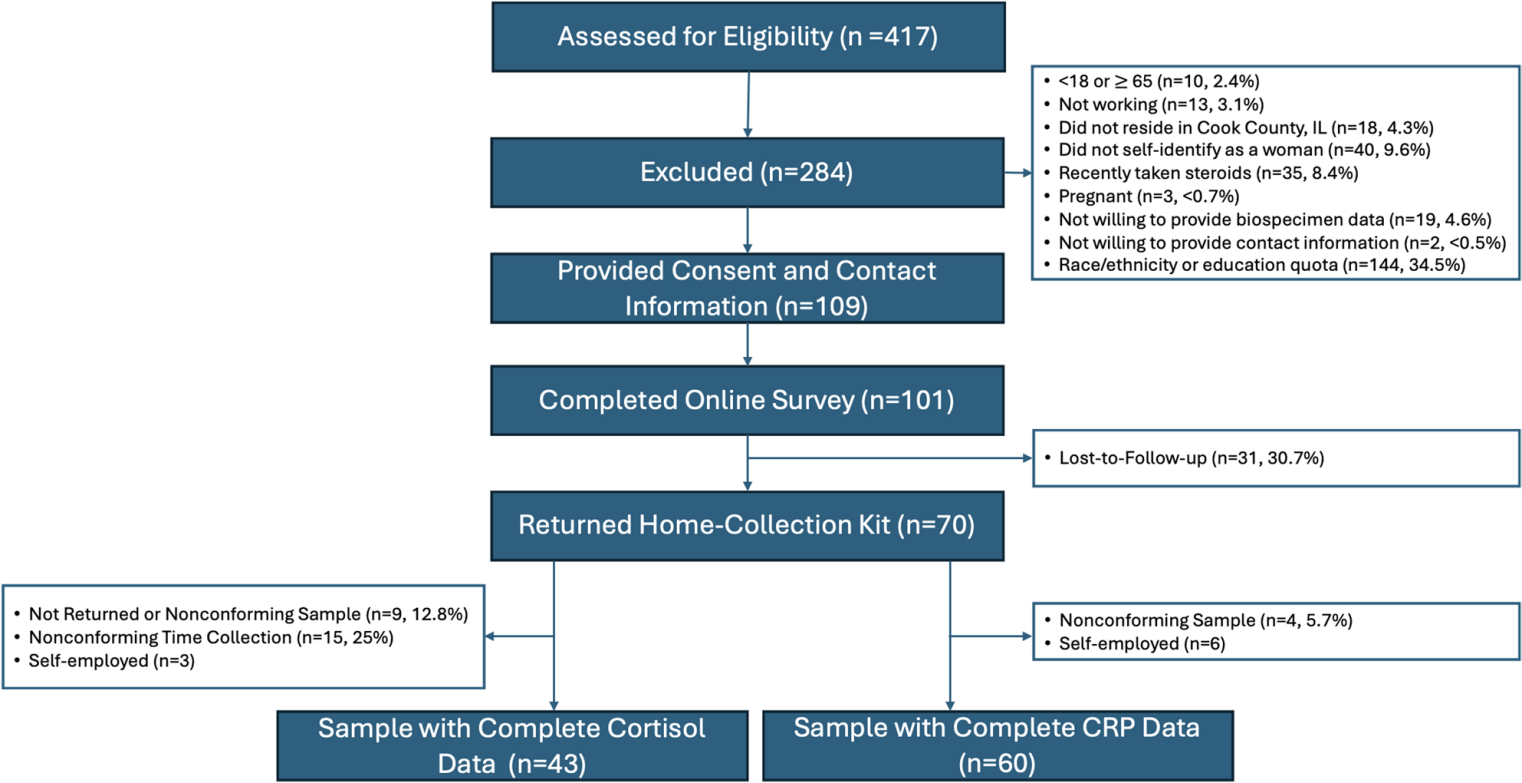
Consort diagram

We reached our recruitment target (*n* = 100) at a rate of ~ 50 women per month, exceeding our anticipated pace. In total, 417 individuals completed the web-based screener; 140 individuals were excluded due to: age (*n* = 10, 2.4%), being unemployed (*n* = 13, 3.1%), not living in Cook County, IL (*n* = 18, 4.3%), not identifying as a woman (*n* = 40, 9.6%), recently taking steroids (*n* = 35, 8.4%), being pregnant (*n* = 3, 0.7%), indicating they would not provide their email and mailing address (*n* = 2, 0.5%), which was required to mail the home-collection kits and provide the incentive, or indicating that they were not willing to provide biospecimen data (*n* = 19, 4.6%) (Fig. 1). An additional 144 individuals were screened out due to the race/ethnicity or education quotas being reached (*n* = 284 total individuals excluded). Of those who qualified based on the eligibility screener (*n* = 133), 109 consented and provided all contact information (name, email, address), 101 then fully completed the online survey, and 70% (*n* = 70) returned the home-collection kits.

### Biospecimen samples

While 70 women returned capillary blood samples, four women (5.7%) returned samples that were not useable due to an insufficient amount of blood provided. The remaining 66 blood samples returned for analyses were in the range of detection for the indicators assayed. Notably, we prioritized the return of the blood samples, as this was the more novel collection measure. For the salivary cortisol, 9 women did not return the samples (i.e., only returned the blood) or returned tubes with low saliva levels. An additional 15 women did not report collection times or reported non-conforming times (e.g., night shift work or time two was before time one) (Fig. 1).

### Participants

A total of 101 women completed the online survey, however, 7 women indicated that they were self-employed, so we were unable to estimate a PES. A majority of the these self-employed women reporting working for themselves (57% versus being a business owner [29%] or subcontractor [14.1%]), 57% had $\:\le\:$highschooldegree, and 57% were Hispanic (see Appendix Table 3).

**Table 3:** Biological Data by Tertile of Precarious Employment^1^

	Most Precarious		Mid Precarious		Least Precarious	
	Mean	SE	Mean	SE	Mean	SE
C-reactive Protein [mg/L]	[*N* = 19]		[*N* = 17]		[*N* = 24]	
	1.1	0.3	1.8	0.5	1.1	0.4
Salivary Cortisol [nmol/L]	[*N* = 16]		[*N* = 12]		[*N* = 15]	
Mean Salivary Cortisol	9.5	1.0	8.6	0.9	8.8	0.8
Mean Wake Up Response	9.5	1.7	10.6	2.0	9.1	1.8
Mean Cortisol Awakening Response	14.1	1.7	13.5	1.9	15.4	1.7
Mean Wake-to-Bed-Slope	-2.1	0.9	-3.9	1.0	-3.9	0.9

*PE * Precarious employment, *SE* Standard Error

^1^ The PES was created using 27 employment indicators and principal components analysis, and then PE tertiles were created

The sample of 94 women with a PES was diverse in age (18–30 years: 27%, 31–40 years: 30%; 41–50 years: 23%; 51–64 years: 20%) and education level ($$\:\le\:$$highschool: 11%, some college: 29%, bachelor’s degree: 40%; graduate degree: 20%), with college-educated individuals being over-represented compared to the population in Chicago (Table 1). We successfully recruited a sample where NH-Black (33%) and Hispanic (36%) women met our quotas and were over-represented compared to the population in Cook County and compared to NH-White women (28%). 55% of the sample reported earning <$40,000, with 26% reporting earning $40,000 - $60,000, 12% reported earning $60,000 - $80,000, and 9% reported earning >$80,000 annually at their primary job. A majority (69%) of our analytic sample resided in a Chicago zipcode. The average PES was 3.6 (SE = 0.3). More than one-third of the sample (36%) were food or nutrition insecure. The mean PSS was 20.4 (SE = 0.6), 56% of participants had severe depressive symptoms and 38% reported they had fair or poor health. The average participant had obesity (BMI: 31.2, SE = 0.8).

Table 1 also compares the characteristics of those who completed the home-collection kit versus those who were lost-to-follow-up. A higher portion of those lost-to-follow-up had lower-education, had lower-income, were more precariously employed, identified as Hispanic, were food and nutrition insecure, and had poorer self-reported health.

Table 2 details selected characteristics and self-reported health by tertile of PE. NH-Black and Hispanic women and women with lower educational attainment were the most precariously employed. A higher proportion of the most precariously employed women (23%) (versus least precarious, 9%) reported experiencing discrimination by race/ethnicity. The most precariously employed women had a higher prevalence of food (58% versus 22% among the least precarious) and nutrition insecurity (45% versus 31% among the least precarious). The most precariously employed women also had poorer self-reported health; 81% reported severe depressive symptoms (versus 41% of least precarious), 52% reported fair or poor health (versus 31%), and BMI (33.9 versus 28.3) and perceived stress (23.7 versus 18.6) were higher, on average, among this group.

**Fig. 2:**
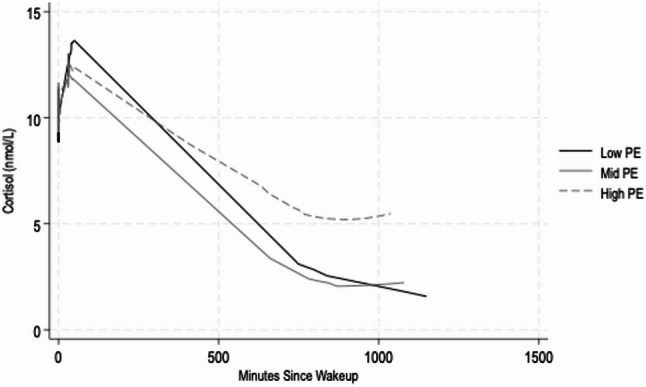
LOESS curve of salivary cortisol by precarious employment tertile

The biological data should be interpreted with caution; the standard errors are large due to small cell sizes per tertile (Table 3) and patterns across PE tertiles were inconsistent, indicating the need for larger study samples. However, there was some suggestive evidence that the most precariously employed group may have lower recovery cortisol trends throughout the day compared to the low and mid precarious groups (Fig. 2).

## Discussion

This study provides preliminary insights into the biological stress profiles of women experiencing varying levels of PE and describes the feasibility of at-home data collection of biospecimen samples among a sample of women, many of whom were precariously employed. In this study, the PES was most strongly influenced by dimensions related to material rewards, work time arrangements, and interpersonal power relations. Participants in this study were enrolled within a relatively short recruitment period (2 months). Completion rates (~ 70% completing the home-collection kit) were promising. In descriptive analyses, the most precariously employed women identified as NH-Black and Hispanic, had lower educational attainment, and a larger proportion reported experiencing employed-related discrimination by race/ethnicity. Precariously employed women also had a higher prevalence of food and nutrition insecurity and had poorer self-reported physical and mental health. Biomarker patterns across PE tertiles were inconsistent but begin to suggest that the highest PE group may have lower recovery cortisol trends throughout the day, compared to the low and mid PE groups.

While this was a pilot study with a small sample, the PES was most strongly influenced by material rewards (e.g., income variability, access to paid time off and benefits), schedule control, and interpersonal power relations (e.g., ability to take leave). These dimensions are consistent with the dimensions that we would hypothesize to be particularly salient for most women in the U.S. labor market, as gendered occupational segregation persists [54, 55] and paid leave is not required at the federal level nor were the rules governing paid leave in Illinois in effect when we collected our data [56]. For example, NH-Black and Hispanic women, are disproportionately represented in service, care, and retail occupations that are marked by low wages, unstable hours, and limited access to benefits and protections. Moreover, women may have more precarious employment because of also navigating delaying labor force entry or re-entry due to pregnancy, childcare, as well as other care responsibilities (e.g., elderly parent); women are 5–8 times more likely than men to have their employment affected by caregiver responsibilities [57], which is consistent with the salience of the questions around their ability to take paid leave. Conceptually, our findings reinforce that PE in the U.S. is not only characterized by economic insecurity, but also by other adverse employment conditions that have implications for women’s health and wellbeing.

Our descriptive findings were generally aligned with our prior research using large nationally representative samples, giving us confidence we can reached our target sample. Similar to our prior research [1, 6, 8] higher proportions of NH-Black and Hispanic women and women with lower educational attainment were the most precariously employed in this sample. A larger proportion of precariously employed women in this sample reported discrimination by race/ethnicity. This is consistent with data suggesting that NH-Black and Black women disproportionately experience employment-related stressors [9–13]. Moreover, prior studies suggest that Black women are more likely to experience disempowerment due to discrimination and/or fear of unfair dismissal [58].

We found that precariously employed women also experienced a number of poor health and well-being outcomes, aligning with prior research [8]. Women experiencing PE had a higher prevalence of food and nutrition insecurity. This is generally consistent with the food insecurity prevalence in Chicago (~ 25% versus the national average, which is ~ 13%) and consistent with limited prior work that finds an association between PE and food insecurity among a convenience sample recruited during the COVID-19 pandemic [59]. Our findings are also consistent with one study that found that nonstandard work, defined as employment that was part-time, varied in hours, or when a worker holds more than one job, was associated with higher food insecurity in a nationally representative sample, which the authors attributed to unstable incomes and more complex schedules [60]. In our sample, 81% of precariously employed women reported severe depressive symptoms, 52% reported fair or poor health and BMI was $\:\ge\:$ 30 kg/m^2^, on average. These results are also consistent with prior U.S.-based studies, which find that precariously employed women had poorer mental [6, 7, 61] and physical [2, 6, 7, 61] health and additional prior research that finds that higher PE is associated with higher BMI among adults [5]. Our descriptive results are also generally consistent with the few prior studies that find an association between worse employment quality, measured using multidimensional indicators, and perceived stress in Spain [62–64] and the U.S [31, 59]. While prior work does support an association between higher PE and higher CRP [31], we observed relatively similar values across PE groups. But these data begin to suggest that precariously employed women have flatter late day declines in salivary cortisol, compared to the low and mid PE group, which is consistent with studies finding NH-Black and Hispanic (versus NH-White) and populations with lower socioeconomic status have less steep declines [65]. However, the biological data should be interpreted with caution, as were not powered to test for differences.

Although at-home biomarker collection is in its infancy, and not without challenges, our study adds to the growing body of literature that suggests at-home collection is largely feasible [38–42], even among what would traditionally be considered a “harder to engage” population because they cannot necessarily be located using rosters or other straightforward methods for reaching workers and/or they have limited time to participate. Specifically for our study, working women, often with families, are less likely to take time off from work to participate in a research study. While alternative recruitment methods are required (e.g., social media), at-home collection can offer a sampling collection process that is lower burden to participants, as it allows participants to respond to survey questions and collect blood specimens at times most convenient to them. Moreover, remote collection allows researchers the opportunity to sample participants in a wider geographic area and recruit a higher sample size in a shorter timeframe. It may also be more appropriate for sensitive questions (e.g., mental health, income) and allow researchers to provide more language options [66].

This study had several limitations. Although the recruitment response was relatively strong, our recruitment methods, particularly the use of a convenient internet sample, led to a bias toward higher educational attainment. Estimates for biological measures are impacted by further selection bias, as disproportionately fewer NH-Black and Hispanic, less-educated, and/or precariously employed participants returned biological specimens. To reduce loss-to-follow-up, future research efforts will consider offering the survey in Spanish and other commonly spoken languages, increasing the incentive as budget feasible, and offering more personalized email and text communication. Additionally, future research will develop a study website and instruction videos, as well as consider alternative collection methods for salivary cortisol, such as the drool method or time-stamped saliva collection tubes, as well as collect nail cortisol to better ensure the accuracy of the biospecimen collection. We provided written instructions, with visuals, for both blood and saliva collection, but additional instructions via video are warranted to further reduce non-conforming samples. While we did ask individuals if racial/ethnic discrimination was a barrier for getting or keeping work, we did not assess whether individuals in the sample had experienced discrimination broadly using a validated scale (e.g., the everyday discrimination scale). Finally, we excluded night shift workers from the analysis because salivary cortisol is highly sensitive to diurnal patterns, which is likely disrupted in those with atypical sleep-wake [67]. As a pilot study, we were not powered to detect significant differences, and a larger study is warranted. Nevertheless, this study offers initial insights on PE and stress and a novel and feasible approach for collection biospecimen data, using multiple biological indicators.

## Conclusions

This pilot study provides preliminary insights into the biological stress profiles of women in varying levels of PE and demonstrates the feasibility of at-home collection of biospecimen samples. Although this was a pilot study, the salience of the material rewards, schedule control, and interpersonal power dimensions begin to provide insights into potential policy levers to reduce precariousness and mitigate stress-related health inequities among women. Future larger-scale studies that are powered to detect associations between PE and biological indicators of stress can build on this work to identify which employment dimensions most strongly predict biological stress among women in diverse occupational settings.

## Supplementary Information

Supplementary Material 1.

## Data Availability

De-identified data are available upon request.

## Appendix

**Appendix Table 1:** Employment Items

	*N*=94	
	N or Mean	% or Standard Error
**Material Rewards**		
**Income**		
<$20,000	26	27.7%
$20,000-39,999	25	26.6%
$40,000-59,999	24	25.5%
$60,000-79,999	11	11.7%
$80,000+	8	8.5%
Not paid if miss work due to illness, family affairs, or personal	36	38.3%
Not usually paid overtime	62	66.0%
Not always paid in full	7	7.4%
**Variation in income**		
Not at all	44	46.8%
A little	21	22.3%
Some	17	18.1%
A lot	8	8.5%
A great deal	4	4.3%
**Portion of cash income**		
none	82	87.2%
< half	4	4.3%
half	1	1.1%
most	7	7.4%
**Fringe benefits **		
No prescription drug plan	65	69.1%
No dental plan	39	41.5%
No health insurance	32	34.0%
No life insurance	46	48.9%
No pension/retirement	41	43.6%
No paid vacation	38	40.4%
No paid sick leave	50	53.2%
**Work Time Arrangements**		
**Paid hours/week**		
0-10	5	5.3%
10-20	1	1.1%
20-30	13	13.8%
30-40	50	53.2%
40-50	18	19.1%
50+	7	7.4%
**Work on call**		
Never	76	80.9%
Some	11	11.7%
Half	3	3.2%
Most	3	3.2%
Always	1	1.1%
**Schedule notice**		
Never	5	5.3%
Some	7	7.4%
Half	4	4.3%
Most	22	23.4%
Always	56	59.6%
**Schedule change**		
Never	20	21.3%
Rarely	35	37.2%
Sometimes	31	33.0%
Often	8	8.5%
**Stability/ Job Type**		
Temp agency	1	1.1%
Seasonal or day worker	2	2.1%
Gig or mobile app worker	1	1.1%
Temporary employee expect to last < 1 year	2	2.1%
Temporary employee expect to last > 1 year	1	1.1%
Permanent part-time, less than 30 hour per week	13	13.8%
Permanent part-time, varied hours (+ or - 30/wk)	12	12.8%
Permanent full-time, consistently 30 hr + week	62	66.0%
**Collective Bargaining **		
Participate in a labor union	16	17.0%
**Interpersonal Relations**		
**Obstacles**		
To take sick leave	71	75.5%
To take doctor leave	75	79.8%
To take vacation leave	68	72.3%
To take family leave	74	78.7%
To take personal leave	72	76.6%
**Comfortable speaking up**		
Never	8	8.5%
Rarely	4	4.3%
Sometimes	33	35.1%
Often	23	24.5%
Always	26	27.7%
**Training and Promotion Opportunities **		
Paid training opportunities	46	48.9%
Good chances for promotion	28	29.8%

**Appendix Table 2:** Factor loadings for precarious employment score

	Factor Loading	Unexplained
Annual income	-0.22	0.55
Paid if miss work due to illness, family affairs, or personal	0.24	0.46
Paid overtime	-0.16	0.77
Not always paid in full	0.11	0.88
Variation in income	0.23	0.51
Portion of cash income	0.12	0.87
Prescription drug plan	0.17	0.73
Dental plan	0.27	0.37
Health insurance	0.27	0.34
Life insurance	0.25	0.44
Pension/retirement	0.27	0.37
Paid vacation	0.28	0.30
Paid sick leave	0.23	0.52
Paid hours/week	0.02	0.99
Work on Call	0.06	0.97
Schedule notice	0.20	0.66
Schedule change	0.15	0.81
Job type	0.12	0.87
Participate in a labor union	-0.05	0.98
Obstacles to take sick leave	0.21	0.62
Obstacles to take doctor leave	0.22	0.55
Obstacles to take vacation leave	0.23	0.53
Obstacles to take family leave	0.23	0.52
Obstacles to take personal leave	0.22	0.56
Comfortable speaking up	0.04	0.99
Paid training opportunities	0.01	0.99
Good chances for promotion	0.12	0.88

**Appendix Table 3:** Selected sample characteristics of the self-employed

	*N*=7	
	N or Mean	% or Standard Error
**Self-Employment Type**		
Business Owner	2	28.6%
Work for Self	4	57.1%
Subcontractor	1	14.3%
**Age**		
18-30	2	28.6%
31-40	1	14.3%
41-50	1	14.3%
51-64	3	42.9%
**Marital Status**		
Married	3	42.9%
Divorced/Separated	2	28.6%
Never Married	2	28.6%
**Education**		
High School	4	57.1%
Some College or associates	1	14.3%
Bachelors	0	0.0%
Graduate	2	28.6%
**Race/Ethnicity**		
Non-Hispanic White	1	14.3%
Non-Hispanic Black	2	28.6%
Non-Hispanic Other	0	0.0%
Hispanic	4	57.1%
**US Born**	5	71.4%
**Nutrition Outcomes**		
Food Insecure^1^	3	42.9%
Nutrition Insecure^2^	3	42.9%
**Self-Reported Health Outcomes**		
Mean Perceived Stress Score^3^	19.0	1.6
Severe Depressive Symptoms^4^	4	57.1%
Fair/Poor Self-Reported Health^5^	2	28.6%
Body Mass Index	28.3	3.7

^1^Food insecurity was defined using the two-item Hunger Vital Sign™ screener. Responses in the affirmative (i.e., often or sometimes, versus never) were assigned a 1 (versus 0)

^2^Nutrition insecurity was queried using the one-item measure developed by the Center for Nutrition & Health Impact. Nutrition insecurity was defined as responding sometimes, often, or always to the question, “In the last 30 days, I worried that the food I was able to eat would hurt my health and well-being.”

^3^Assessed using the 10-item Cohen’s Perceived Stress Scale (PSS, possible range 0-50)

^4^Assessed using the Center for Epidemiologic Studies Depression Scale (possible range 0-40); depression severity was dichotomized as no or mild (0–9) versus moderate or severe (10–40)

^5^Assessed using the one-item question “in general, would you say your health is” with the response items including excellent, very good, good, fair, or poor; self-reported health was dichotomized as excellent, very good, good (=0) versus fair or poor (=1)

## Abbreviations

BMI: Body mass index
CESD: Center for Epidemiologic Studies Depression Scale
CAR: Cortisol awakening response
CRP: C-reactive protein
PSS: Perceived Stress Scale
PE: Precarious Employment
PES: Precarious Employment Score
SRH: Self-reported health
U.S: United States

## References

[1] Oddo VM, Zhuang CC, Andrea SB, Eisenberg-Guyot J, Peckham T, Jacoby D, et al. Changes in precarious employment in the United States: A longitudinal analysis. Scand J Work Environ Health. 2021;47(3):171–80. 33283874 10.5271/sjweh.3939 PMC8126438

[2] Peckham TK, Fujishiro K, Hajat A, Flaherty BP, Seixas N. Evaluating employment quality as a determinant of health in a changing labor market. RSF Russell Sage Found J Soc Sci. 2019;5(4):258–81. 10.7758/RSF.2019.5.4.09 PMC6756794 31548990

[3] Benach J, Vives A, Amable M, Vanroelen C, Tarafa G, Muntaner C. Precarious employment: understanding an emerging social determinant of health. Annu Rev Public Health. 2014;35:229–53. 10.1146/annurev-publhealth-032013-182500 24641559

[4] Kreshpaj B, Orellana C, Burström B, Davis L, Hemmingsson T, Johansson G, et al. What is precarious employment? A systematic review of definitions and operationalizations from quantitative and qualitative studies. Scand J Work Environ Health. 2020;46(3):235–47. 31901944 10.5271/sjweh.3875

[5] Oddo VM, Zhuang CC, Dugan JA, Andrea SB, Hajat A, Peckham T, et al. Association between precarious employment and BMI in the United States. Obesity. 2023;31(1):234–42. 36541156 10.1002/oby.23591 PMC9782712

[6] Andrea SB, Eisenberg-Guyot J, Oddo VM, Peckham T, Jacoby D, Hajat A. Beyond Hours Worked and Dollars Earned: Multidimensional EQ, Retirement Trajectories and Health in Later Life. Work Aging Retire. 2021;8(1):51–73. 35035984 10.1093/workar/waab012 PMC8742984

[7] Eisenberg-Guyot J, Peckham T, Andrea SB, Oddo V, Seixas N, Hajat A. Life-course trajectories of employment quality and health in the US: A multichannel sequence analysis. Soc Sci Med. 2020;264:113327. 32919256 10.1016/j.socscimed.2020.113327 PMC7607590

[8] Hajat A, Andrea SB, Oddo VM, Winkler MR, Ahonen EQ. Ramifications of precarious employment for health and health inequity: Emerging trends from the Americas. Annu Rev Public Health. 2024;45. 10.1146/annurev-publhealth-071321-042437 PMC11128534 38012123

[9] Quillian L, Lee JJ, Oliver M. Evidence from field experiments in hiring shows substantial additional racial discrimination after the callback. Soc Forces. 2020;99(2):732–59.

[10] Bureau of Labor Statistics. Median usual weekly earnings of full-time wage and salary workers by selected characteristics. [Internet]. 2022 Apr. Report No.: USDL-22-0624. Available from: https://www.bls.gov/news.release/pdf/wkyeng.pdf

[11] Mandel H, Semyonov M. The growing racial pay gap is linked to rising income inequality and continued occupational segregation and discrimination [Internet]. 2016 [cited 25 Nov 5]. Available from: https://blogs.lse.ac.uk/usappblog/2016

[12] Mandel H, Semyonov M. Going back in time? Gender differences in trends and sources of the racial pay gap, 1970 to 2010. Am Sociol Rev. 2016;81(5):1039–68.

[13] Kristal T, Cohen Y, Navot E. Benefit inequality among American workers by gender, race, and ethnicity, 1982–2015. Sociol Sci. 2018;5:461–88.

[14] Schneider D, Harknett K. It’s about time: How work schedule instability matters for workers, families, and racial inequality [Internet]. 2019 [cited 25 Nov 5]. Available from: https://shift.hks.harvard.edu/files/2019/10/Its-About-Time-How-Work-Schedule-Instability-Matters-for-Workers-Families-and-Racial-Inequality.pdf

[15] Segerstrom SC, Miller GE. Psychological stress and the human immune system: A meta-analytic study of 30 years of inquiry. Psychol Bull. 2004;130(4):601–30. 15250815 10.1037/0033-2909.130.4.601 PMC1361287

[16] Won E, Kim YK. Stress, the autonomic nervous system, and the immune-kynurenine pathway in the etiology of depression. Curr Neuropharmacol. 2016;14(7):665–73. 27640517 10.2174/1570159X14666151208113006 PMC5050399

[17] Miller GE, Chen E, Zhou ES. If it goes up, must it come down? Chronic stress and the hypothalamic-pituitary-adrenocortical axis in humans. Psychol Bull. 2007;133(1):25. 17201569 10.1037/0033-2909.133.1.25

[18] Björntorp P. Do stress reactions cause abdominal obesity and comorbidities? Obes Rev. 2001;2(2):73–86. 12119665 10.1046/j.1467-789x.2001.00027.x

[19] Whitworth JA, Williamson PM, Mangos G, Kelly JJ. Cardiovascular consequences of cortisol excess. Vasc Health Risk Manag. 2005;1(4):291–9. 17315601 10.2147/vhrm.2005.1.4.291 PMC1993964

[20] Phillips DIW, Barker DJP, Fall CHD, Seckl JR, Whorwood CB, Wood PJ, et al. Elevated plasma cortisol concentrations: a link between low birth weight and the insulin resistance syndrome? J Clin Endocrinol Metab. 1998;83(3):757–60. 9506721 10.1210/jcem.83.3.4634

[21] Reynolds RM, Walker BR. Human insulin resistance: the role of glucocorticoids. Diabetes Obes Metab. 2003;5(1):5–12. 12542720 10.1046/j.1463-1326.2003.00221.x

[22] Knight JM, Avery EF, Janssen I, Powell LH. Cortisol and depressive symptoms in a population-based cohort of midlife women. Psychosom Med. 2010;72(9):855–61. 20841562 10.1097/PSY.0b013e3181f4ab87 PMC3115732

[23] Liu YZ, Wang YX, Jiang CL. Inflammation: the common pathway of stress-related diseases. Front Hum Neurosci. 2017;11:316. 28676747 10.3389/fnhum.2017.00316 PMC5476783

[24] Bourassa KJ, Caspi A, Brennan GM, Hall KS, Harrington H, Houts R, et al. Which types of stress are associated with accelerated biological aging? Comparing perceived stress, stressful life events, childhood adversity, and posttraumatic stress disorder. Psychosom Med. 2023;85(5):389–96. 37053097 10.1097/PSY.0000000000001197 PMC10239326

[25] Ammous F, Zhao W, Ratliff SM, Mosley TH, Bielak LF, Zhou X, et al. Epigenetic age acceleration is associated with cardiometabolic risk factors and clinical cardiovascular disease risk scores in African Americans. Clin Epigenetics. 2021;13:1–13. 33726838 10.1186/s13148-021-01035-3 PMC7962278

[26] Lu AT, Quach A, Wilson JG, Reiner AP, Aviv A, Raj K, et al. DNA methylation GrimAge strongly predicts lifespan and healthspan. Aging. 2019;11(2):303. 30669119 10.18632/aging.101684 PMC6366976

[27] Maddock J, Castillo-Fernandez J, Wong A, Cooper R, Richards M, Ong KK, et al. DNA methylation age and physical and cognitive aging. J Gerontol Ser A. 2020;75(3):504–11. 10.1093/gerona/glz246 PMC8414926 31630156

[28] Brisson D, McCune S, Wilson JH, Speer SR, McCrae JS, Hoops Calhoun K. A systematic review of the association between poverty and biomarkers of toxic stress. J Evid-Based Soc Work. 2020;17(6):696–713. 10.1080/26408066.2020.1769786 32657246

[29] Serwinski B, Salavecz G, Kirschbaum C, Steptoe A. Associations between hair cortisol concentration, income, income dynamics and status incongruity in healthy middle-aged women. Psychoneuroendocrinology. 2016;67:182–8. 26923848 10.1016/j.psyneuen.2016.02.008 PMC4821175

[30] Samuel L, Szanton SL, Fedarko NS, Simonsick EM. Leveraging naturally occurring variation in financial stress to examine associations with inflammatory burden among older adults. J Epidemiol Community Health. 2020;74(11):892–7. 32665370 10.1136/jech-2020-213807 PMC7580025

[31] Oddo VM, Mabrouk S, Andrea SB, Ahonen EQ, Winkler MR, Vignola EF, et al. The association between precarious employment and stress among working aged individuals in the United States. Prev Med. 2024;187:108123. 39216552 10.1016/j.ypmed.2024.108123 PMC11700481

[32] George S, Duran N, Norris K. A systematic review of barriers and facilitators to minority research participation among African Americans, Latinos, Asian Americans, and Pacific Islanders. Am J Public Health. 2014;104(2):e16–31. 24328648 10.2105/AJPH.2013.301706 PMC3935672

[33] Ford JG, Howerton MW, Lai GY, Gary TL, Bolen S, Gibbons MC, et al. Barriers to recruiting underrepresented populations to cancer clinical trials: a systematic review. Cancer Interdiscip Int J Am Cancer Soc. 2008;112(2):228–42. 10.1002/cncr.23157 18008363

[34] Branson RD, Davis K Jr, Butler KL. African Americans’ participation in clinical research: importance, barriers, and solutions. Am J Surg. 2007;193(1):32–9. 17188084 10.1016/j.amjsurg.2005.11.007

[35] Haley SJ, Southwick LE, Parikh NS, Rivera J, Farrar-Edwards D, Boden-Albala B. Barriers and strategies for recruitment of racial and ethnic minorities: perspectives from neurological clinical research coordinators. J Racial Ethn Health Disparities. 2017;4:1225–36. 28176157 10.1007/s40615-016-0332-y PMC5547022

[36] McDermott MM, Newman AB. Remote research and clinical trial integrity during and after the coronavirus pandemic. JAMA. 2021;325(19):1935–6. 33885728 10.1001/jama.2021.4609

[37] Waddell CJ, Pellegrini GJ, Persad N, Filardo TD, Prasad N, Carson WC, et al. Minimally invasive blood collection for an mpox serosurvey among people experiencing homelessness. J Appl Lab Med. 2024;9(5):1014–9. 38842196 10.1093/jalm/jfae035 PMC11869119

[38] Koenig MR, Wesselink AK, Kuriyama AS, Chaiyasarikul A, Hatch EE, Wise LA. Feasibility of mail-based biospecimen collection in an online preconception cohort study. Front Reprod Health. 2023;4:1052231. 36699143 10.3389/frph.2022.1052231 PMC9869415

[39] Haack AJ, Lim FY, Kennedy DS, Day JH, Adams KN, Lee JJ, et al. home RNA: A self-sampling kit for the collection of peripheral blood and stabilization of RNA. Anal Chem. 2021;93(39):13196–203. 34546711 10.1021/acs.analchem.1c02008 PMC9134895

[40] Kalish H, Klumpp-Thomas C, Hunsberger S, Baus HA, Fay MP, Siripong N, et al. Undiagnosed SARS-CoV-2 seropositivity during the first 6 months of the COVID-19 pandemic in the United States. Sci Transl Med. 2021;13(601):eabh3826. 34158410 10.1126/scitranslmed.abh3826 PMC8432952

[41] Fuller G, Njune Mouapi K, Joung S, Shufelt C, van den Broek I, Lopez M, et al. Feasibility of patient-centric remote dried blood sampling: the prediction, risk, and evaluation of major adverse cardiac events (PRE-MACE) study. Biodemography Soc Biol. 2020;65(4):313–22. 10.1080/19485565.2020.1765735 PMC8464354 33243027

[42] Koulman A, Rennie KL, Parkington D, Tyrrell CS, Catt M, Gkrania-Klotsas E, et al. The development, validation and application of remote blood sample collection in telehealth programmes. J Telemed Telecare. 2024;30(4):731–8. 35538704 10.1177/1357633X221093434 PMC11027437

[43] Delahaye L, Veenhof H, Koch BC, Alffenaar JWC, Linden R, Stove C. Alternative sampling devices to collect dried blood microsamples: state-of-the-art. Ther Drug Monit. 2021;43(3):310–21. 33470777 10.1097/FTD.0000000000000864

[44] Poverty and Employment Precocity in Southern Ontario. Employment Precarity Index [Internet]. 2014 [ cited 25 Nov 5]. Available from: https://pepso.ca/tools

[45] Calloway EE, Carpenter LR, Gargano T, Sharp JL, Yaroch AL. Development of new measures to assess household nutrition security, and choice in dietary characteristics. Appetite. 2022;179:106288. 36049571 10.1016/j.appet.2022.106288

[46] Cohen S, Williamson G. Perceived stress in a probability sample of the US. In The Social Psychology of Health. Spacapam, S. Oskamp, S. Eds. Sage Publications, Inc. 1988:31–67.

[47] Cohen S, Janicki-Deverts D. Who’s stressed? Distributions of psychological stress in the United States in probability samples from 1983, 2006, and 2009 1. J Appl Soc Psychol. 2012;42(6):1320–34.

[48] Andresen EM, Malmgren JA, Carter WB, Patrick DL. Screening for depression in well older adults: evaluation of. Prev Med. 1994;10:77–84. 8037935

[49] Marsland AL, Walsh C, Lockwood K, John-Henderson NA. The effects of acute psychological stress on circulating and stimulated inflammatory markers: a systematic review and meta-analysis. Brain Behav Immun. 2017;64:208–19. 28089638 10.1016/j.bbi.2017.01.011 PMC5553449

[50] Steptoe A, Hamer M, Chida Y. The effects of acute psychological stress on circulating inflammatory factors in humans: a review and meta-analysis. Brain Behav Immun. 2007;21(7):901–12. 17475444 10.1016/j.bbi.2007.03.011

[51] Julià M, Vanroelen C, Bosmans K, Van Aerden K, Benach J. Precarious Employment and Quality of Employment in Relation to Health and Well-being in Europe. Int J Health Serv. 2017;47(3):389–409. 28449605 10.1177/0020731417707491

[52] Van Aerden K, Moors G, Levecque K, Vanroelen C. Measuring employment arrangements in the European labour force: a typological approach. Soc Indic Res. 2014;116(3):771–91.

[53] Whitehead AL, Sully BG, Campbell MJ. Pilot and feasibility studies: is there a difference from each other and from a randomised controlled trial? Contemp Clin Trials. 2014;38(1):130–3. 24735841 10.1016/j.cct.2014.04.001

[54] Fujishiro K, Ahonen EQ, Winkler M. Poor-quality employment and health: How a welfare regime typology with a gender lens Illuminates a different work-health relationship for men and women. Soc Sci Med. 2021;291:114484. 34656919 10.1016/j.socscimed.2021.114484 PMC8671289

[55] Homan P. Structural sexism and health in the United States: A new perspective on health inequality and the gender system. Am Sociol Rev. 2019;84(3):486–516.

[56] Illinois Department of Labor. Paid Leave for All Workers Act [Internet]. 2024 [cited 2025 Nov 5]. Available from: https://labor.illinois.gov/laws-rules/paidleave.html

[57] Almeida B, Salas-Betsch B. Fact sheet: The state of women in the labor market in 2023 [Internet]. 2023 [cited 2025 Nov 5] Available from: https://www.americanprogress.org/article/fact-sheet-the-state-of-women-in-the-labor-market-in-2023/

[58] Parker K, Funk C. Gender discrimination comes in many forms for today’s working women [Internet]. 2017 [cited 25 Nov 5]. Available from: https://pewresearch.org

[59] Oddo VM, Jones-Smith JC, Knox MA. Changes in Precarious Employment and Health in the United States Amidst the COVID-19 Pandemic. Prev Med Rep. 2023;31:102113. 36688136 10.1016/j.pmedr.2023.102113 PMC9841738

[60] Coleman-Jensen AJ. Working for peanuts: nonstandard work and food insecurity across household structure. J Fam Econ Issues. 2011;32:84–97.

[61] Bhattacharya A, Ray T. Precarious work, job stress, and health-related quality of life. Am J Ind Med. 2021;64(4):310–9. 33543533 10.1002/ajim.23223 PMC9904539

[62] Belvis F, Bolíbar M, Benach J, Julià M. Precarious Employment and Chronic Stress: Do Social Support Networks Matter? Int J Environ Res Public Health. 2022;19(3):1909. 35162929 10.3390/ijerph19031909 PMC8835513

[63] Caroz-Armayones JM, Benach J, Delclós C, Julià M. The double burden of precariousness: linking housing, employment, and perceived stress–a cross-sectional study. Int J Environ Health Res. 2023;33(11):1102–11. 35549954 10.1080/09603123.2022.2075330

[64] Julià M, Méndez-Rivero F, Gómez-Gómez Á, Pozo ÓJ, Bolíbar M. Association between Precarious Employment and Chronic Stress: Effect of Gender, Stress Measurement and Precariousness Dimensions—A Cross-Sectional Study. Int J Environ Res Public Health. 2022;19(15):9099. 35897463 10.3390/ijerph19159099 PMC9330896

[65] Hajat A, Diez-Roux A, Franklin TG, Seeman T, Shrager S, Ranjit N, et al. Socioeconomic and race/ethnic differences in daily salivary cortisol profiles: the multi-ethnic study of atherosclerosis. Psychoneuroendocrinology. 2010;35(6):932–43. 20116177 10.1016/j.psyneuen.2009.12.009 PMC2875317

[66] Hensen B, Mackworth-Young C, Simwinga M, Abdelmagid N, Banda J, Mavodza C, et al. Remote data collection for public health research in a COVID-19 era: ethical implications, challenges and opportunities. Health Policy Plan. 2021;36(3):360–8. 33881138 10.1093/heapol/czaa158 PMC7928874

[67] Grosser L, Knayfati S, Yates C, Dorrian J, Banks S. Cortisol and shiftwork: A scoping review. Sleep Med Rev. 2022;64:101581. 35872400 10.1016/j.smrv.2021.101581

