# Supplementary material for: A feasibility study exploring precarious employment and stress-related health among women
Source: BMC Public Health. 2026 Apr 15;26:1556. doi: 10.1186/s12889-026-27366-5 (PMC13173993; doi:10.1186/s12889-026-27366-5)
Supplement: Supplementary file 1 — Supplementary Material 1. [file 12889_2026_27366_MOESM1_ESM.docx]

| **Appendix Table 1. Employment Items** | | |
| --- | --- | --- |
|  | N=94 | |
|  | N or Mean | % or Standard Error |
| **Material Rewards** |  |  |
| **Income** |  |  |
| <$20,000 | 26 | 27.7% |
| $20,000-39,999 | 25 | 26.6% |
| $40,000-59,999 | 24 | 25.5% |
| $60,000-79,999 | 11 | 11.7% |
| $80,000+ | 8 | 8.5% |
| Not paid if miss work due to illness, family affairs, or personal | 36 | 38.3% |
| Not usually paid overtime | 62 | 66.0% |
| Not always paid in full | 7 | 7.4% |
| **Variation in income** |  |  |
| Not at all | 44 | 46.8% |
| A little | 21 | 22.3% |
| Some | 17 | 18.1% |
| A lot | 8 | 8.5% |
| A great deal | 4 | 4.3% |
| **Portion of cash income** |  |  |
| none | 82 | 87.2% |
| < half | 4 | 4.3% |
| half | 1 | 1.1% |
| most | 7 | 7.4% |
| **Fringe benefits** |  |  |
| No prescription drug plan | 65 | 69.1% |
| No dental plan | 39 | 41.5% |
| No health insurance | 32 | 34.0% |
| No life insurance | 46 | 48.9% |
| No pension/retirement | 41 | 43.6% |
| No paid vacation | 38 | 40.4% |
| No paid sick leave | 50 | 53.2% |
| **Work Time Arrangements** |  |  |
| **Paid hours/week** |  |  |
| 0-10 | 5 | 5.3% |
| 10-20 | 1 | 1.1% |
| 20-30 | 13 | 13.8% |
| 30-40 | 50 | 53.2% |
| 40-50 | 18 | 19.1% |
| 50+ | 7 | 7.4% |
| **Work on call** |  |  |
| Never | 76 | 80.9% |
| Some | 11 | 11.7% |
| Half | 3 | 3.2% |
| Most | 3 | 3.2% |
| Always | 1 | 1.1% |
| **Schedule notice** |  |  |
| Never | 5 | 5.3% |
| Some | 7 | 7.4% |
| Half | 4 | 4.3% |
| Most | 22 | 23.4% |
| Always | 56 | 59.6% |
| **Schedule change** |  |  |
| Never | 20 | 21.3% |
| Rarely | 35 | 37.2% |
| Sometimes | 31 | 33.0% |
| Often | 8 | 8.5% |
| **Stability/ Job Type** |  |  |
| Temp agency | 1 | 1.1% |
| Seasonal or day worker | 2 | 2.1% |
| Gig or mobile app worker | 1 | 1.1% |
| Temporary employee expect to last < 1 year | 2 | 2.1% |
| Temporary employee expect to last > 1 year | 1 | 1.1% |
| Permanent part-time, less than 30 hour per week | 13 | 13.8% |
| Permanent part-time, varied hours (+ or - 30/wk) | 12 | 12.8% |
| Permanent full-time, consistently 30 hr + week | 62 | 66.0% |
| **Collective Bargaining** |  |  |
| Participate in a labor union | 16 | 17.0% |
| **Interpersonal Relations** |  |  |
| **Obstacles** |  |  |
| To take sick leave | 71 | 75.5% |
| To take doctor leave | 75 | 79.8% |
| To take vacation leave | 68 | 72.3% |
| To take family leave | 74 | 78.7% |
| To take personal leave | 72 | 76.6% |
| **Comfortable speaking up** |  |  |
| Never | 8 | 8.5% |
| Rarely | 4 | 4.3% |
| Sometimes | 33 | 35.1% |
| Often | 23 | 24.5% |
| Always | 26 | 27.7% |
| **Training and Promotion Opportunities** |  |  |
| Paid training opportunities | 46 | 48.9% |
| Good chances for promotion | 28 | 29.8% |

| **Appendix Table 2. Factor Loadings for Precarious Employment Score** | | |
| --- | --- | --- |
|  | Factor Loading | Unexplained |
| Annual income | -0.22 | 0.55 |
| Paid if miss work due to illness, family affairs, or personal | 0.24 | 0.46 |
| Paid overtime | -0.16 | 0.77 |
| Not always paid in full | 0.11 | 0.88 |
| Variation in income | 0.23 | 0.51 |
| Portion of cash income | 0.12 | 0.87 |
| Prescription drug plan | 0.17 | 0.73 |
| Dental plan | 0.27 | 0.37 |
| Health insurance | 0.27 | 0.34 |
| Life insurance | 0.25 | 0.44 |
| Pension/retirement | 0.27 | 0.37 |
| Paid vacation | 0.28 | 0.30 |
| Paid sick leave | 0.23 | 0.52 |
| Paid hours/week | 0.02 | 0.99 |
| Work on Call | 0.06 | 0.97 |
| Schedule notice | 0.20 | 0.66 |
| Schedule change | 0.15 | 0.81 |
| Job type | 0.12 | 0.87 |
| Participate in a labor union | -0.05 | 0.98 |
| Obstacles to take sick leave | 0.21 | 0.62 |
| Obstacles to take doctor leave | 0.22 | 0.55 |
| Obstacles to take vacation leave | 0.23 | 0.53 |
| Obstacles to take family leave | 0.23 | 0.52 |
| Obstacles to take personal leave | 0.22 | 0.56 |
| Comfortable speaking up | 0.04 | 0.99 |
| Paid training opportunities | 0.01 | 0.99 |
| Good chances for promotion | 0.12 | 0.88 |

| **Appendix Table 3. Selected Sample Characteristics of the Self-Employed** | | |
| --- | --- | --- |
|  | N=7 | |
|  | N or Mean | % or Standard Error |
| **Self-Employment Type** |  |  |
| Business Owner | 2 | 28.6% |
| Work for Self | 4 | 57.1% |
| Subcontractor | 1 | 14.3% |
| **Age** |  |  |
| 18-30 | 2 | 28.6% |
| 31-40 | 1 | 14.3% |
| 41-50 | 1 | 14.3% |
| 51-64 | 3 | 42.9% |
| **Marital Status** |  |  |
| Married | 3 | 42.9% |
| Divorced/Separated | 2 | 28.6% |
| Never Married | 2 | 28.6% |
| **Education** |  |  |
| $\leq$ High School | 4 | 57.1% |
| Some College or associates | 1 | 14.3% |
| Bachelors | 0 | 0.0% |
| Graduate | 2 | 28.6% |
| **Race/Ethnicity** |  |  |
| Non-Hispanic White | 1 | 14.3% |
| Non-Hispanic Black | 2 | 28.6% |
| Non-Hispanic Other | 0 | 0.0% |
| Hispanic | 4 | 57.1% |
| **US Born** | 5 | 71.4% |
| **Nutrition Outcomes** |  |  |
| Food Insecure^1^ | 3 | 42.9% |
| Nutrition Insecure^2^ | 3 | 42.9% |
| **Self-Reported Health Outcomes** |  |  |
| Mean Perceived Stress Score^3^ | 19.0 | 1.6 |
| Severe Depressive Symptoms^4^ | 4 | 57.1% |
| Fair/Poor Self-Reported Health^5^ | 2 | 28.6% |
| Body Mass Index | 28.3 | 3.7 |

^1^ Food insecurity was defined using the two-item Hunger Vital Sign™ screener. Responses in the affirmative (i.e., often or sometimes, versus never) were assigned a 1 (versus 0).

^2^ Nutrition insecurity was queried using the one-item measure developed by the Center for Nutrition & Health Impact. Nutrition insecurity was defined as responding sometimes, often, or always to the question, “In the last 30 days, I worried that the food I was able to eat would hurt my health and well-being.”

^3^ Assessed using the 10-item Cohen’s Perceived Stress Scale (PSS, possible range 0-50).

^4^ Assessed using the Center for Epidemiologic Studies Depression Scale (possible range 0-40); depression severity was dichotomized as no or mild (0–9) versus moderate or severe (10–40).

^5^ Assessed using the one-item question “in general, would you say your health is” with the response items including excellent, very good, good, fair, or poor; self-reported health was dichotomized as excellent, very good, good (=0) versus fair or poor (=1).
